# Allogeneic Hematopoietic Cell Transplantation for Therapy-Related Myeloid Leukemia following Orthotopic Cardiac Transplantation

**DOI:** 10.1155/2013/140138

**Published:** 2013-03-30

**Authors:** Richard J. Lin, Richard A. Larson, Koen van Besien, Elizabeth S. Rich

**Affiliations:** ^1^Department of Medicine, Weill Cornell Medical College, New York, NY 10065, USA; ^2^Section of Hematology/Oncology, Department of Medicine and the Cancer Research Center, University of Chicago, Chicago, IL 60637, USA; ^3^Department of Medicine, Rush University Medical Center, 1725 W. Harrison Street, Ste 836, Chicago, IL 60612, USA

## Abstract

Therapy-related myeloid neoplasm (t-MN) is a subtype of acute myeloid leukemia with adverse cytogenetics and poor overall prognosis despite intensive induction chemotherapy and allogeneic hematopoietic cell transplantation (allo-HCT). It is increasingly recognized as a late complication of chronic immunosuppression in patients who have received solid organ transplantation. In this paper, we describe a case of t-MN following orthotopic cardiac transplantation and its treatment with allo-HCT. We discuss molecular and biological challenges and considerations in double solid organ and bone marrow transplantation and review similar cases at our institution. Our experience suggests general feasibility and safety of allo-HCT in patients who have received solid organ transplantation.

## 1. Case Presentation

A 52-year-old Caucasian man with a history of hypertension and insulin-dependent type 2 diabetes mellitus underwent orthotopic cardiac transplantation for ischemic cardiomyopathy. Subsequent immunosuppression consisted of cyclosporine A (CsA), mycophenolate mofetil (MMF), and low dose prednisone. Four years later, he presented with a 3-week history of sinus congestion, fever, chills, and non-productive cough. Further workup showed leukocytosis of 137,000/*μ*L with 31% blasts and 63% promonocytes, hemoglobin of 7.0 g/dL, platelets of 55,000/*μ*L, and acute kidney injury with creatinine of 3.3 mg/dL. A bone marrow biopsy revealed monoblastic therapy-related myeloid neoplasm (t-MN). The karyotype was 46XY, inv(9)(p11q13)c, t(10; 11)(p11.2; q23) [100%]. Inv(9) is a recognized human constitutional (c) polymorphism of unknown phenotypic significance while t(10; 11) is a recurring cytogenetic abnormality in acute myeloid leukemia [1]. There was no evidence of the *FLT3* gene internal tandem deletion or D835 mutation, another common adverse prognostic factor in acute myeloid leukemia [1]. MMF was discontinued. CsA was changed to tacrolimus, and low dose prednisone was continued. The patient was initially treated with hydroxyurea for leukoreduction, and his hospital course was complicated by pulmonary hemorrhage requiring mechanical ventilation, tumor lysis syndrome, and complicated parapneumonic pleural effusion. He then underwent induction chemotherapy with high dose cytarabine (2000 mg/m^2^) and mitoxantrone (20 mg/m^2^). A morphologic and cytogenetic complete remission (CR1) was achieved three months later (Table 1). 

Allogeneic hematopoietic cell transplantation (allo-HCT) was planned, as the patient's only sibling was an 8 of 8 HLA-match with the same ABO blood group A. Moreover, there were no anti-donor blood group antibodies in his serum. However, allo-HCT was delayed by vancomycin-resistant enterococcus empyema that required thoracotomy with decortication. Cardiac biopsy at that time showed no evidence of acute rejection. Unfortunately, while recuperating from surgery, the patient's t-MN relapsed. He was treated again with high dose cytarabine (2000 mg/m^2^) and mitoxantrone (20 mg/m^2^). At day 13 bone marrow biopsy showed a severely hypocellular marrow (3%) with scattered residual blasts (10–15%). The patient underwent a non-myeloablative allo-HCT following conditioning with fludarabine, melphalan, and alemtuzumab 8 months after the diagnosis of t-MN [2]. His post-transplant course was uncomplicated; myeloid cells engrafted by day +13 and platelets by day +27. He continued to receive tacrolimus and low dose prednisone for immunosuppression of his cardiac graft. On day +27, engraftment analysis revealed that donor DNA accounted for > 95% of total DNA in the unfractionated marrow sample and 88(±3)% of DNA in the CD3(+) population. On day +145, a complete morphologic and cytogenetic remission (CR2) was documented. There were no signs of rejection of the patient's cardiac graft, and his cardiac function remained normal with a left ventricular ejection fraction of 59%. However, 4 months after allo-HCT, the patient presented with diplopia and was found to have central nervous system relapse of t-MN. He underwent intrathecal chemotherapy and whole brain radiation therapy with symptomatic improvement. Systemic chemotherapy with cytarabine and etoposide was started, but the patient's hospital course was complicated by sepsis, and he died from multi-organ failure (Table 1).

## 2. Discussion

Non-Hodgkin lymphoma is the most common malignancy that occurs after solid organ transplantation [3, 4], but t-MN has also been described [5–8]. T-MN generally portends a poor prognosis despite treatment with induction chemotherapy and, more recently, allo-HCT [9]. Treatment of t-MN in solid organ transplant recipients is even more challenging as these patients require ongoing immunosuppression and often have multiple co-morbidities. This patient's treatment course illustrates several important issues in considering allo-HCT for t-MN following solid organ transplantation and in conducting post-transplant care.

First, the patient's cadaveric cardiac graft was an 8 of 8 HLA-mismatch with the patient, initially raising the question of whether allo-HCT might result in rejection of the patient's transplanted heart. Fortunately, his sibling donor was a full HLA-match as well as ABO-compatible, the major determinant in cardiac transplant, thus reducing the risk of rejecting the patient's cardiac graft. Second, we screened the recipient's serum and found no preformed HLA antibodies against the transplanted heart. A right heart biopsy was negative for acute cellular rejection, and a C4d stain of the biopsy, a marker for allograft rejection, was also negative. Finally, although there was the potential for a T-cell mediated immune response by the stem cell donor's lymphocytes against the previously transplanted heart, we saw no sign of this during the peri-transplant period. A literature search found several previous case reports of HCT in recipients of cardiac transplantation [10–13]. All but one patient survived HCT without rejecting their cardiac graft. However, the peri-transplant mortality and morbidity was high, reflecting the fine balance between risks of serious infections and the need for ongoing immunosuppression [10–13]. 

Interestingly, t-MN is increasingly observed in patients receiving long-term immunosuppression for both solid organ transplants and chronic inflammatory diseases [14]. Immunosuppressants such as CsA and MMF have been implicated, and the use of azathioprine has been postulated to affect the host cell's DNA mismatch repair system leading to t-MN [14]. A review of the t-MN database at the University of Chicago identified five patients who developed t-MN after solid organ transplantation (Table 2). The median age was 56 years (range, 50–58); median latency following transplantation was 7 years (range, 4.3–12.3); and median survival from diagnosis of t-MN was 1.5 years (range, 1–3.6). Four of five patients received CsA, two received MMF, and two received azathioprine. Karyotype was normal in two patients, del(7q) in two patients, and t(10; 11) in the last patient. Del(7q) and t(10; 11) are two recurring, poor-risk cytogenetic abnormalities observed in t-MN [9]. Three of five patients, including the patient presented here, underwent HCT (2 allogeneic and 1 autologous) without rejecting transplanted solid organs. Overall, our experience suggests that HCT may be performed safely to treat t-MN developing in solid organ transplant recipients receiving concurrent immunosuppressants.

## Figures and Tables

**Table 1:** Clinical course and cytogenetic abnormalities of the patient.

Time	0	3 months	6 months	8 months	12 months	12 months
Clinical course	Acute Leukemia	Induction chemo	Relapse	Reinduction chemo + allo-HCT	CNS relapse	Systemic relapse
Treatment	Hydroxyurea	Cytarabine + mitoxantrone		Cytarabine + mitoxantrone + allo-HCT (Flu/Mel/alemtuzumab)	Intrathecal methotrexate + hydrocortisone; cranial radiation	Cytarabine + Etoposide
Bone marrow biopsy	t-AML, 91% blasts	CR1	Recurrent t-AML, 53% blasts	CR2		Recurrent t-AML, 40% blasts
Karyotype	46XY, inv(9)c, t(10;11)	46XY, inv(9)c	46XY, inv(9)c, 20%; 46XY, inv(9)c, t(10;11), 75%	46XY, 100% donor		46XY, inv(9)c, t(10;11)

**Table 2:** Post-solid organ transplant t-MNs at the University of Chicago.

	Patient 1	Patient 2	Patient 3	Patient 4	Patient 5
Age/sex (years)	57/M	56/M	50/M	58/F	55/F
Organ transplant	Heart	Kidney	Heart	Liver	Kidney
Immuno-suppressive therapy	CsA MMF Prednisone	CsA MMF Prednisone	CsA azathioprine Prednisone	CsA azathioprine Prednisone	Cytoxan Prednisone
Latency (years)	4.3	4.4	12.3	7.7	7
WHO classification	t-AML	t-AML	t-MDS	t-MDS	t-MDS
Treatment	Induction chemo + allo-HCT	Induction chemo + allo-HCT	Induction chemo + auto-HCT	Induction chemo	Supportive care
Survival (years)	1	1.5	3.6	1.3	1.9
Karyotype	46XY, inv(9)c, t(10;11)	Normal	46XY, 55%; 46XY, del 7, 25%; 46XY, del 11, 20%	Normal	46XX, 55%; 46XX, inv(9)c, del 7, 41%; 46XX, inv(9)c, del 19, 4%
